# Electronic, Vibrational, and Structural Properties of the Natural Mineral Ferberite (FeWO_4_): A High-Pressure Study

**DOI:** 10.1021/acs.inorgchem.4c00345

**Published:** 2024-03-30

**Authors:** Daniel Diaz-Anichtchenko, Jesus E. Aviles-Coronado, Sinhué López-Moreno, Robin Turnbull, Francisco J. Manjón, Catalin Popescu, Daniel Errandonea

**Affiliations:** † Departamento de Física Aplicada-ICMUV, MALTA Consolider Team, Universidad de Valencia, Dr. Moliner 50, Burjassot, 46100 Valencia, Spain; ‡ División de Materiales Avanzados, IPICYT, Camino a la Presa de San José 2055 Col. Lomas 4a sección, San Luis Potosi 78216, Mexico; § CONAHCYT—División de Materiales Avanzados, IPICYT, Camino a la Presa de San José 2055 Col. Lomas 4a sección, San Luis Potosi 78216, Mexico; ∥ Grupo de Ciencia e Ingeniería Computacionales—Centro Nacional de Supercómputo, IPICYT, Camino a la Presa de San José 2055 Col. Lomas 4a sección, San Luis Potosi 78216, Mexico; ⊥ Instituto de Diseño para la Fabricación y Producción Automatizada, MALTA Consolider Team, Universitat Politècnica de València, Camí de Vera s/n, 46022 València, Spain; # CELLS-ALBA Synchrotron Light Facility, Cerdanyola 08290, Barcelona, Spain

## Abstract

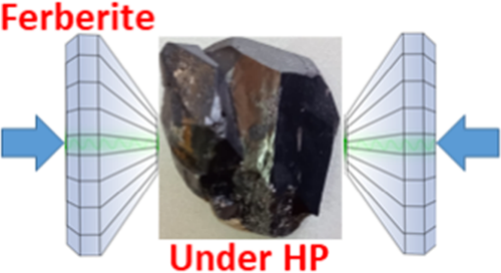

This paper reports an experimental high-pressure study of natural mineral ferberite (FeWO_4_) up to 20 GPa using diamond-anvil cells. First-principles calculations have been used to support and complement the results of the experimental techniques. X-ray diffraction patterns show that FeWO_4_ crystallizes in the wolframite structure at ambient pressure and is stable over a wide pressure range, as is the case for other wolframite AWO_4_ (A = Mg, Mn, Co, Ni, Zn, or Cd) compounds. No structural phase transitions were observed for FeWO_4_, in the pressure range investigated. The bulk modulus (*B*_0_ = 136(3) GPa) obtained from the equation of state is very close to the recently reported value for CoWO_4_ (131(3) GPa). According to our optical absorption measurements, FeWO_4_ has an indirect band gap that decreases from 2.00(5) eV at ambient pressure to 1.56(5) eV at 16 GPa. First-principles simulations yield three infrared-active phonons, which soften with pressure, in contrast to the Raman-active phonons. These results agree with Raman spectroscopy experiments on FeWO_4_ and are similar to those previously reported for MgWO_4_. Our results on FeWO_4_ are also compared to previous results on other wolframite-type compounds.

## Abstract 2

A systematic study of the high-pressure behavior of iron tungstate is reported. A combination of density functional theory calculations, X-ray diffraction, Raman spectroscopy, and optical absorption experiments has been used to determine the effect of pressure on the structural, vibrational, and electronic properties of iron tungstate. A comparison between present results and previous results from related tungstates is also made.

## 1 Introduction

Wolframite-type AWO_4_ compounds (A = Mg, Mn, Fe, Co, Ni, Zn, Cd) form an interesting class of bimetallic oxides because of their properties,^1,2^ including the magnetic properties for A = Fe, Co, and Ni,^3^ and even multiferroic properties in the case of MnWO_4_.^4^^4^ The magnetism is dominated by partially filled 3d orbitals of the divalent *A* cations.^5^ In addition to interesting magnetic properties, wolframites, such as FeWO_4_, have been used to develop supercapacitors and photocatalytic and photoluminescent materials.^6−8^ FeWO_4_ can be used for the cheap and environmentally friendly production of ammonia^9^ and for applications in phase-change optical recording devices.^10^ Therefore, accurate knowledge of the physical properties of this material is essential for its technological applications.

FeWO_4_ is one of the less studied AWO_4_ wolframites.^1^ Curiously, most studies on this material have focused on nanoparticles,^11,12^ with single crystals mainly synthesized to characterize their magnetic properties.^10^ FeWO_4_ showed an antiferromagnetic behavior at low temperatures with a Néel temperature of 75 K.^10^ In particular, the characterization of the band gap energy at ambient conditions has been conducted on compacted powders using diffuse reflectance^10,12−14^ rather than accurate optical absorption measurements on single crystals.^15^ As a consequence, the reported values of the band gap energy range from 1.8 to 2.2 eV. Additionally, there are previous Raman studies at ambient pressure, but not all of the expected Raman-active modes have been measured in these studies.^2,11,16^ On the other hand, nothing is known about the compressibility and structural stability of FeWO_4_ under high-pressure (HP) conditions.

**Figure 1:**
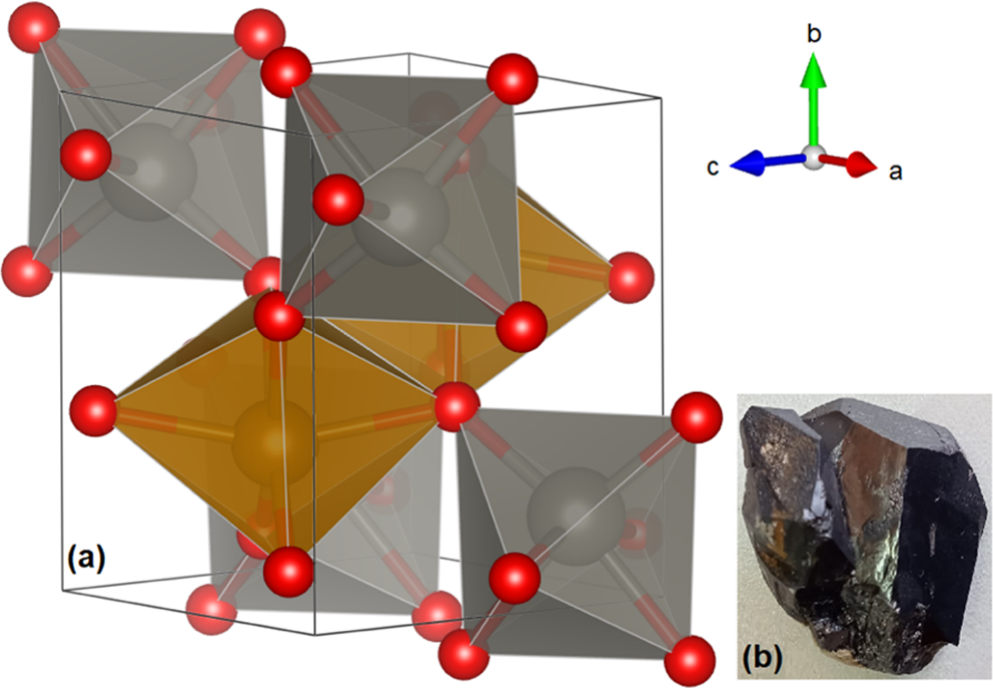
(a) Crystal structure of the mineral ferberite (FeWO_4_). FeO_6_ octahedra are shown in brown, and WO_6_ octahedra are shown in gray. The oxygen atoms are shown in red. The black solid lines represent the unit cell. The arrows indicate the crystal axes. (b) Photograph of the ferberite crystal used for experiments.

The crystal structure of FeWO_4_ is monoclinic (space group *P*2/*c*) and contains two formula units per unit cell.^17^ The structure is shown in Figure 1a. The metal ions (Fe^2+^ and W^6+^) occupy half of the octahedral holes in a slightly deformed hexagonal close-packed lattice of oxygen atoms. The crystal structure can be described as two zigzag chains of edge-sharing FeO_6_ or WO_6_ octahedral units running along [001]. The high-spin d^6^ electronic configuration of Fe^2+^ distorts the FeO_6_ octahedron due to the Jahn–Teller effect.^18^

It is well known that pressure can drastically change the interatomic distances in solids, which in turn can lead to a variety of fascinating phenomena such as metallization,^19^ superconductivity,^20^ changes in chemical bonding,^21^ and the formation of new compounds.^22^ Wolframites have been studied under compression over the past decade by several research groups.^1^ Due to their bulk modulus, an external pressure of 10 GPa causes a 10% change in the unit-cell volume. Consequently, the electronic, magnetic, vibrational, and elastic properties can be dramatically altered.^23,24^ However, it is known that several AWO_4_ wolframite-type compounds do not undergo phase transitions up to pressures of around 20 GPa.^1,2,7,23−25^

In the stability range of the wolframite structure, compression is anisotropic, with the symmetry of the structure decreasing with increasing pressure.^23,24^ The structural changes cause a decrease in the electronic band gap energy of NiWO_4_, MnWO_4_, and CoWO_4_,^5,23,26^ which has been attributed to an increase in hybridization between the 3d electrons of the divalent A cation and the 2p electrons of the oxygen atoms. However, the influence of the pressure on the properties of FeWO_4_ has not been investigated. The influence of the pressure on the crystal structure of FeWO_4_ has also not been studied experimentally. Therefore, we considered it timely to conduct a study of the HP behavior of FeWO_4_ and to investigate the Raman spectrum and optical absorption of single crystals of FeWO_4_ in detail. Here, we report optical absorption, Raman, and X-ray diffraction (XRD) measurements at ambient pressure and HP in a natural crystal of the mineral ferberite (FeWO_4_). The experimental results are complemented by first-principles calculations.

## 2 Experimental Details

The experiments were performed on samples obtained from natural ferberite crystals provided by Fabre Minerals from the Monte Cambillaya mining district, La Paz, Bolivia. An image of the original crystal is shown in Figure 1b. The dimensions of the natural crystal were 2.9 cm × 2.4 cm × 1.3 cm. Electron microprobe analysis was performed to determine the impurities present in the natural crystal. Nb (0.06%) and Ta (0.02%) were the only detected impurities. Such a minimal concentration of impurities is not expected to affect the properties studied in this work. Optical absorption and Raman experiments were performed on 40 μm × 40 μm × 10 μm platelets oriented perpendicular to the cleavage plane (010).^10^ The color of the crystals was dark brown in transmitted light. Powder XRD measurements were performed on a finely ground powder from a fragment of the original single crystal. All experiments at HP were performed using a membrane-driven diamond-anvil cell with diamond culets of 500 μm in diameter. Stainless steel gaskets preindented to a thickness of 55 μm were used. The pressure-transmitting medium was a 4:1 methanol–ethanol mixture, which provides quasi-hydrostatic conditions up to 10 GPa.^27^ Pressure was measured using ruby fluorescence with an error of less than 0.05 GPa.^28^

Synchrotron powder XRD experiments were performed at the BL04-MSPD beamline of ALBA synchrotron^29^ using a monochromatic X-ray beam with a wavelength of 0.4642 Å. The X-ray beam was focused down to a 20 μm × 20 μm spot. XRD was collected with a Rayonix SX165 charge-coupled device (CCD) image plate. The two-dimensional (2D) patterns were integrated using FIT2D,^30^ and FullProf was used to analyze (Rietveld refinement) the integrated 1D XRD patterns.^31^ Raman experiments were performed using an inVia Renishaw Raman spectrometer system with a 5× magnification objective. A laser wavelength of 682 nm with an output of less than 10 mW was used to avoid sample heating. The spectral resolution was greater than 2 cm^–1^. The optical absorption experiments were carried out in visible (Vis)–near-infrared (NIR) range using an optical setup consisting of a halogen lamp, reflecting optical objectives, and a Vis–NIR Ocean Optics spectrometer.^32^ The optical absorption was calculated by dividing the transmittance spectrum of the sample in normal incidence by the spectrum of the reference source.

## 3 Computational Details

First-principles calculations were performed within the framework of the density functional theory (DFT)^33^ and the projector-augmented wave (PAW)^34,35^ method as implemented in the Vienna Ab initio Simulation Package (VASP).^36^ A plane-wave energy cutoff of 520 eV was used to ensure high precision in calculations. The exchange-correlation energy was described within the generalized gradient approximation (GGA) in the GGA + *U* method with the Perdew–Burke–Ernzerhof for solids (PBEsol) functional^37^ to account for the strong correlation between the electrons in the d shell based on the method developed by Dudarev.^38^ In this method, Coulomb Interaction *U* and onsite exchange interaction *J*_H_ are treated together as *U*_eff_ = *U* – *J*_H_. For our GGA + *U* calculations, we chose *U* = 6 eV and *J*_H_ = 0.95 eV. Similar values were previously used with success in the study of other iron and ABO_4_ compounds.^39−42^ All properties computed in this study were calculated under the GGA + *U* approach. For the calculations, we considered nonmagnetic, ferromagnetic, and antiferromagnetic configurations. We found that for the pressures covered by this study, the configuration with the lowest energy is the antiferromagnetic one. All physical properties simulated in this work were calculated for this configuration.

The Monkhorst–Pack scheme^43^ was employed to discretize the Brillouin zone (BZ) integrations with a mesh 4 × 4 × 4, which corresponds to a set of 16 special *k*-points in the irreducible BZ for the wolframite structure. In the relaxed equilibrium configuration, the forces are less than 1 meV/Å per atom in each Cartesian direction. The highly converged results on forces are required to calculate the dynamical matrix using the direct force constant approach.^44^ This allows us to identify the irreducible representation and the character of the phonon modes at the zone center (Γ point). The electronic structure was obtained by using the primitive cell with a larger set of *k*-points.

## 4 Results and Discussion

### 4.1 XRD Measurements

**Figure 2:**
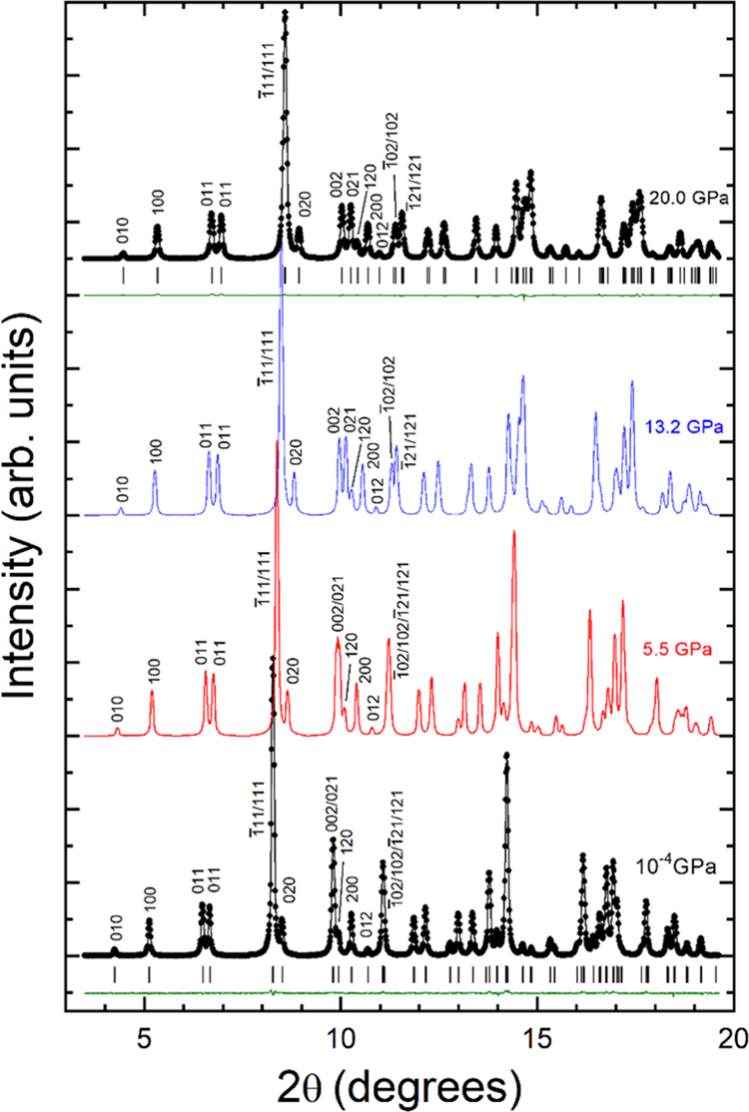
XRD patterns measured in natural mineral ferberite (FeWO_4_) at selected pressures. At ambient pressure (bottom, 10^–4^ GPa) and the highest pressure (top, 20.0 GPa), experimental data are shown with black symbols, Rietveld refinements with black lines, and residuals with green lines. Vertical ticks are the calculated positions for reflections. The Miller indices of the low-angle peaks are indicated.

Figure 2 shows powder XRD patterns of mineral ferberite (FeWO_4_) measured at selected pressures. The bottom trace of the figure shows the results at ambient pressure together with the results from the Rietveld refinement performed using the structural model reported in the literature.^17^ All measured peaks can be explained by the wolframite-type structure (space group *P*2/*c*).^17^ The goodness-of-fit parameters of the refinement are *R*_WP_ = 4.16%, *R*_P_ = 2.82%, and χ^2^ = 1.39, supporting a good agreement of the model with the experimental data. In Table 1, we can observe the good agreement between our structural parameters obtained at ambient pressure, those reported in the literature^17^ and those obtained from our DFT calculations.

**Table 1:** Unit-Cell Parameters (*a*, *b*, *c*, and β), Volume (*V*), and Atomic Positions of Natural Mineral Ferberite (FeWO_4_) Obtained from the Rietveld Refinements at Ambient Pressure^a^

	exp.	theo.	ref (17)
*a* (Å)	4.742(2)	4.7142	4.730(3)
*b* (Å)	5.722(2)	5.7369	5.703(2)
*c* (Å)	4.971(2)	4.9512	4.952(2)
β (deg)	90.14(6)	90.513	90.0(2)
*V* (Å^3^)	134.9(2)	133.9	133.6(4)

atom	site	exp.	theo.	ref (17)
Fe	2f	(0.5, 0.3234(5), 0.25)	(0.5, 0.32182, 0.25)	(0.5, 0.3256, 0.25)
W	2e	(0, 0.1854(3), 0.25)	(0, 0.16597, 0.25)	(0, 0.1799, 0.25)
O1	4g	(0.2158(9), 0.1049(9), 0.5775(9))	(0.21576, 0.10476, 0.56913)	(0.2159, 0.1050, 0.5660)
O2	4g	(0.2584(9), 0.3836(9), 0.0958(9))	(0.25518, 0.37562, 0.10428)	(0.2538, 0.3744, 0.1096)

a They are compared with results from present calculations and previous experiments.^17^

Figure 2 shows that the only change induced by pressure in the powder XRD pattern is the shift of peaks toward higher angles due to the contraction of the lattice parameters. We did not detect the emergence of any extra peaks up to 20 GPa. In fact, all XRD patterns up to this pressure can be properly refined assuming the same structural model used for the ambient pressure results. This is illustrated in Figure 2 by the Rietveld refinement of the XRD pattern measured at 20 GPa, which is the highest pressure measured. At this pressure, we obtained *R*_WP_ = 6.33%, *R*_P_ = 3.98%, and χ^2^ = 1.98, thereby confirming the assignment of the wolframite-type structure. Therefore, we can conclude that, as in other wolframites,^11^ there is no phase transition in FeWO_4_ up to 20 GPa.

Two important additional observations can be made from the XRD patterns. The first one is that we did not observe any significant peak broadening beyond the quasi-hydrostatic limit of the pressure medium (10 GPa).^31^ This suggests that the influence of nonhydrostatic stresses is negligible in our experiments. The second one is the splitting of several peaks as the pressure increases, which can be seen in Figure 2 by following the evolution of the 002/021 peaks and 1̅02/102/1̅21/121 peaks, which are close to 8 and 11°, respectively. This fact indicates that the compression is not isotropic, as we show next.

**Figure 3:**
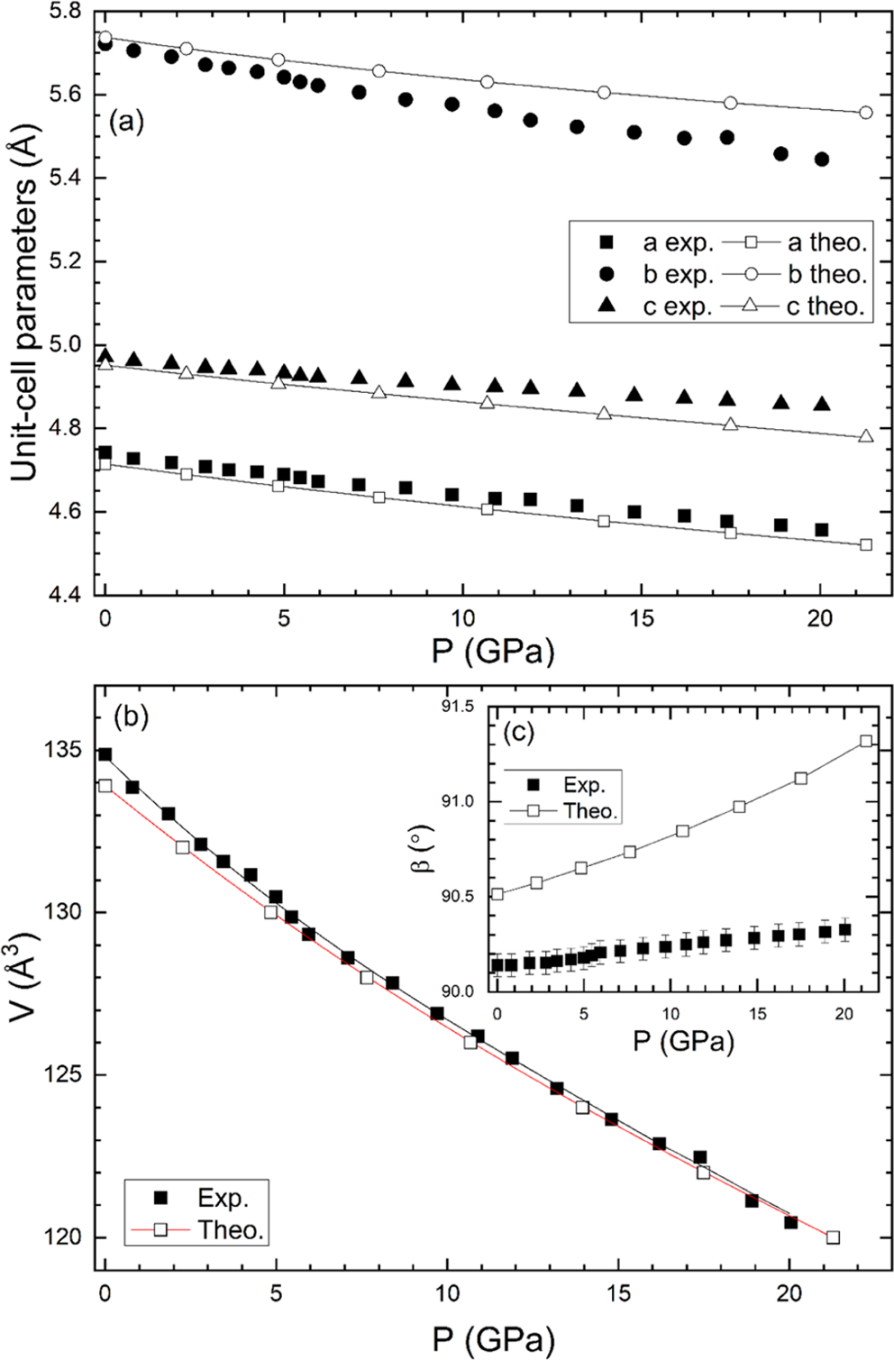
Pressure dependence of (a) lattice parameters *a*, *b*, and *c*, (b) unit-cell volume *V*, and (c) angle β of FeWO_4_. Solid (empty) symbols are results obtained from experiments (calculations). (b) Equations of state obtained from experiments (black line) and calculations (red line).

From the XRD results, the pressure dependence of the unit-cell parameters is obtained and compared with the results from the calculations (see Figure 3). Both experiments and calculations show a similar pressure dependence for the lattice parameters of the crystal structure. The only slight difference between calculations and experiments is that according to calculations the unit-cell parameters are slightly less compressible than in experiments. Such discrepancies are within the typical discrepancies between experiments and computational results in other wolframites.^23^ It might be possible to overcome them using different exchange-correlation functionals in calculations, but a systematic study of the optimum functional for FeWO_4_ is beyond the scope of this study. Our results confirm that the compression of FeWO_4_ is slightly anisotropic. In particular, the *b* parameter is the most compressible lattice parameter, and the *c* parameter is the least compressible, as is the case for CdWO_4_, MgWO_4_, and MnWO_4_ according to single-crystal XRD measurements.^45^ This result is confirmed by the fact that the Miller indices with *k* ≠ 0 move faster with pressure than those with *k* = 0.

In Figure 3, it can be seen that according to the XRD data, there is an increase in the β angle with pressure, which also favors the aforementioned peak splitting described above. In experiments, two different trends are observed below and above 5 GPa. However, the slope change in the behavior of β at 5 GPa is within the experimental uncertainties, which suggests that the two different trends in the behavior of the β angle can be an experimental artifact. In fact, such a slope change is not observed in calculations (see the inset of Figure 3). Calculations predict a smooth behavior for the β angle, with a steeper increase than in experiments. A similar difference is observed when comparing the behavior of the β angle obtained from DFT calculations and XRD experiments in NiWO_4_.^23^

**Figure 4:**
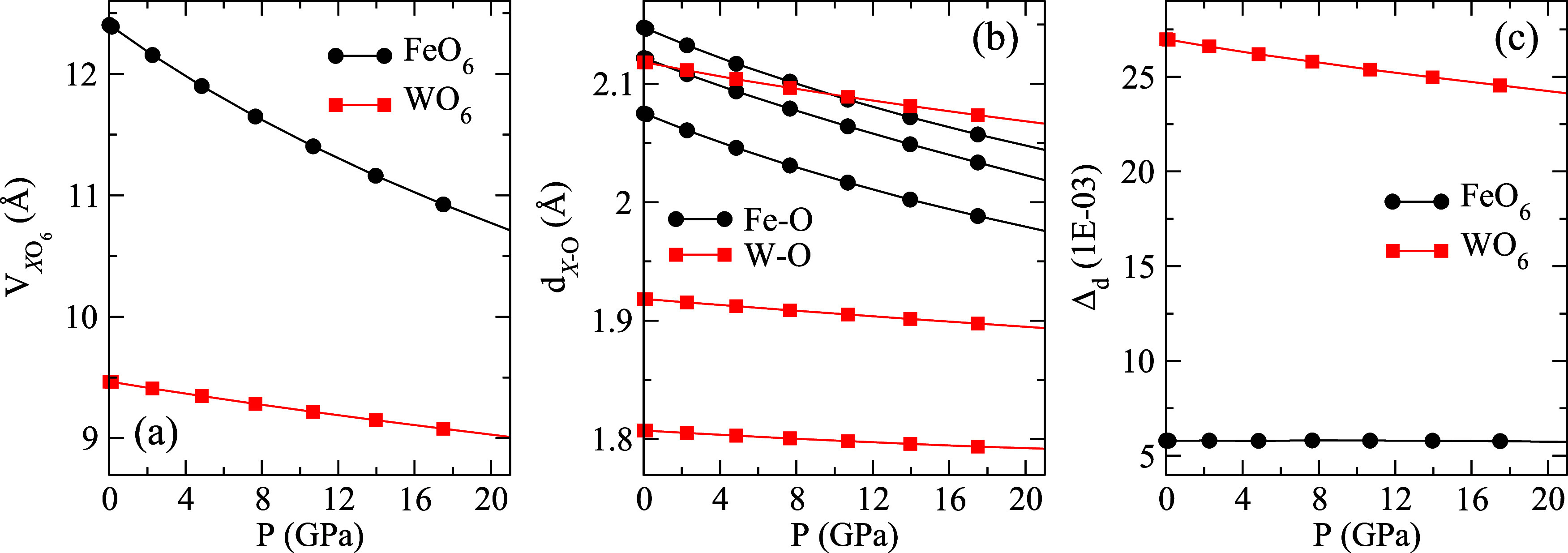
Pressure dependence of the computed (a) polyhedral volume, (b) interatomic distances *d*_Fe–O_ and *d*_W–O_, and (c) distortion index Δ_d_ in FeWO_4_.

As shown in Figure 4a, the computed polyhedral volume of the distorted FeO_6_ octahedra compresses faster than that of the WO_6_ octahedra. This result is due to the stronger compression of the Fe–O distances (*d*_Fe–O_) than the W–O distances (*d*_W–O_) (see Figure 4b). Note that W–O bonds are stronger (more ionic) than Fe–O bonds in terms of the Laplacian of the charge density at the respective bond critical point (∇^2^ρ).^46,47^ This is the reason behind the stiffness of W–O bonds in comparison to that of Fe–O bonds. On the other hand, Figure 4c shows that the polyhedral distortion index Δ_d_, calculated using the definition established by Baur,^48^ decreases with pressure for the WO_6_ octahedra, but remains almost constant for FeO_6_. In Figure 4c, it can be seen that up to 20 GPa, the Jahn–Teller distortion is not suppressed by pressure effects. The fact that FeO_6_ octahedra are more compressible than WO_6_ octahedra can be correlated with the slightly anisotropic compressibility of FeWO_4_. The wolframite structure is formed by chains of FeO_6_ and WO_6_ octahedra, which alternate along the *b*-axis, and thus the change in volume of the FeO_6_ octahedra favors the compressibility along the *b*-axis rather than along the other axes.^45^ On the other hand, the fact that zigzag chains of WO_6_ octahedra run along the *c*-axis makes it the least compressible one.

Since the crystal structure of FeWO_4_ is monoclinic, the compressibility tensor is not diagonal, and a proper description of the compressibility of the material is obtained from the eigenvalues and eigenvectors of the compressibility tensor.^49^ We obtained these from our experiments using PASCal.^50^ The results are reported in Table 2. Among the principal axes of compressibility, the most compressible one is parallel to the *b*-axis, in agreement with our previous conclusions on axial compressibility.

**Table 2:** Eigenvalues, λ*_i_*, and Eigenvectors, *e*_ν_*_i_*, of the Isothermal Compressibility Tensor of FeWO_4_

λ_1_ = 2.33(5) × 10^–3^ Gpa^–1^	*e*_ν1_ = (0,1,0)
λ_2_ = 1.92(2) × 10^–3^ Gpa^–1^	*e*_ν2_ = (10,0,1)
λ_3_ = 1.09(1) 10^–3^ Gpa^–1^	*e*_ν3_ = (1,0,–10)

The results for the pressure dependence of the volume were fitted with a third-order Birch–Murnaghan equation of state^51^ using the program EoSFit.^52^ The obtained bulk modulus (*B*_0_), its pressure derivative (*B*_0_), and the volume (*V*_0_) at zero pressure are given in Table 3. The bulk modulus determined from our experiments, *B*_0_ = 136(2) GPa, agrees within two standard deviations with the bulk modulus previously reported for the rest of the *A*WO_4_ wolframites.^1^ The calculated bulk modulus is 10% larger than that determined from experiments. Such a difference is within the typical range of discrepancies between DFT calculations and experiments. A detailed discussion of the reason for this discrepancy can be found in ref (53).

**Table 3:** Unit-Cell Volume (*V*_0_), Bulk Modulus (*B*_0_), and Bulk Modulus Pressure Derivative (*B*_0_′) at Zero Pressure of FeWO_4_ Determined Using a Third-Order Birch–Murnaghan EOS^a^

	*V*_0_ (Å^3^)	*B*_0_ (GPa)	*B*_0_^′^
experiment	134.9(5)	136(2)	5.0(4)
theoretical	133.9(1)	150(1)	4.8(2)

a We present results from experiments and calculations.

### 4.2 Raman Measurements

According to group-theory analysis, FeWO_4_ has 36 vibrational modes at the Γ point of the Brillouin zone: Γ = 8A_g_ + 10B_g_ + 8A_u_ + 10B_u_. Three vibrations correspond to acoustic modes (A_u_ + 2B_u_); the rest are optical modes. This means that 18 Raman-active modes (8A_g_ + 10B_g_) and 15 infrared (IR)-active modes (7A_u_ + 8B_u_) are expected. To the best of our knowledge, no IR experiments have been reported on FeWO_4_. Regarding the Raman-active modes, only seven modes were reported for FeWO_4_ in refs (11) and (16). In contrast, 15 were reported for FeWO_4_ in ref (2). The nondetection of all Raman modes in previous studies was due to mainly single-crystal orientation or overlapped peaks.

**Figure 5:**
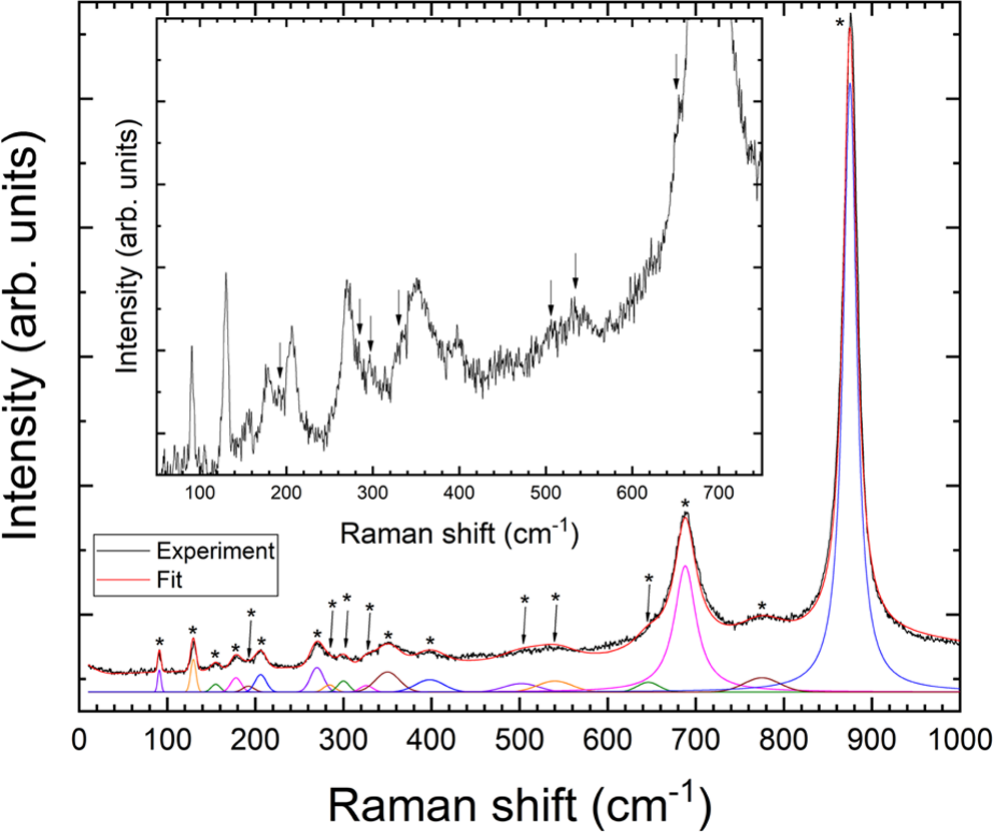
Raman spectrum of the natural mineral ferberite (FeWO_4_) measured at ambient conditions in a sample before loading it into the diamond-anvil cell. The black line corresponds to the experimental spectrum. The red line represents the overall fit. The contribution of each phonon to the fit is shown in different colors in the lower part of the figure. The 18 phonons are identified by asterisks (*). The arrows identify the weakest modes. The inset shows a zoom of the 50–750 cm^–1^ region to facilitate the identification of these peaks.

In Figure 5, we show the Raman spectrum of the natural ferberite mineral (FeWO_4_) at ambient pressure together with the fit we made, assuming peaks with a Lorentzian shape. The symmetry assignment of each Raman mode has been done with the theoretical results. Note that our Raman spectrum is similar to that reported for the mineral ferberite in the RRUFF database.^54^ In our measurements, we detected the 18 Raman-active modes predicted for FeWO_4_. Eleven modes can be directly visualized in Figure 5. The other seven modes are indicated by arrows in the figure. To clearly show the other, we have included a zoom of the 50–750 cm^–1^ region of the Raman spectrum. In this zoom, the seven weakest modes are also identified by arrows.^55^

The values obtained for all Raman modes of FeWO_4_ are summarized in Table 4. They compare well to those previously reported.^2^ The good match obtained with the Lorentzian multipeak fitting analysis used for the deconvolution of the different modes and the comparison with previous studies make us confident in our mode identification. The modes at 192, 300, and 350 cm^–1^ were never reported before. The difference in wavenumber between this work and ref (2) is within 5 cm^–1^ (the accuracy of both setups is 2 cm^–1^). This could be due to an offset in the calibrations between the two setups and/or to the fact that the present study was carried out in a natural mineral and the previous study was performed in a synthetic sample.

**Table 4:** Wavenumbers of Raman-Active Modes in FeWO_4_ at Ambient Pressure^a^

mode	ω_exp_ (cm^–1^), this work	ω_DFT_ (cm^–1^), this work	ω_exp_ (cm^–1^), ref (2)	ω_DFT_ (cm^–1^), ref (2)
B_g_	91	97.8	86	92
A_g_	129	130.9	124	132
B_g_	155	159	154	162
B_g_	178	183.7	174	179
B_g_	192	194.9		184
A_g_	206	222.9	208	213
B_g_	271	275.5	266	263
A_g_	284	279.1	299	278
B_g_	300	301.1		295
A_g_	328	333.1	330	330
B_g_	350	350.2		350
A_g_	398	393.7	401	406
B_g_	502	502.1	500	483
A_g_	539	536.3	534	530
B_g_	648	644.1	653	637
A_g_	688	686.8	692	676
B_g_	775	764.4	777	754
A_g_	875	869.2	878	866

a Results from the present work are compared with those reported in the literature.^2^

Regarding calculations, the present results agree slightly better with our experiments than those of previous investigations.^2^ This difference could be mainly due to the exchange-correlation functional and the slight difference in the *U*_eff_ parameter. The relative difference between the present calculations and experiments is within 5%, which can be considered quite good.^54^Figure 4 shows that the strongest modes are in the highest-frequency region, corresponding to two A_g_ modes that can be assigned to internal symmetric vibrations of the WO_6_ octahedron. Not surprisingly, the strongest mode, which is assigned to the symmetric stretching vibration, in FeWO_4_ has nearly the same frequency (875 cm^–1^) as in MnWO_4_ (887.5 cm^–1^), NiWO_4_ (881 cm^–1^), and CoWO_4_ (887 cm^–1^).^2^ This is because the W–O distances and, hence, the force constants are nearly identical in the four compounds.

**Figure 6:**
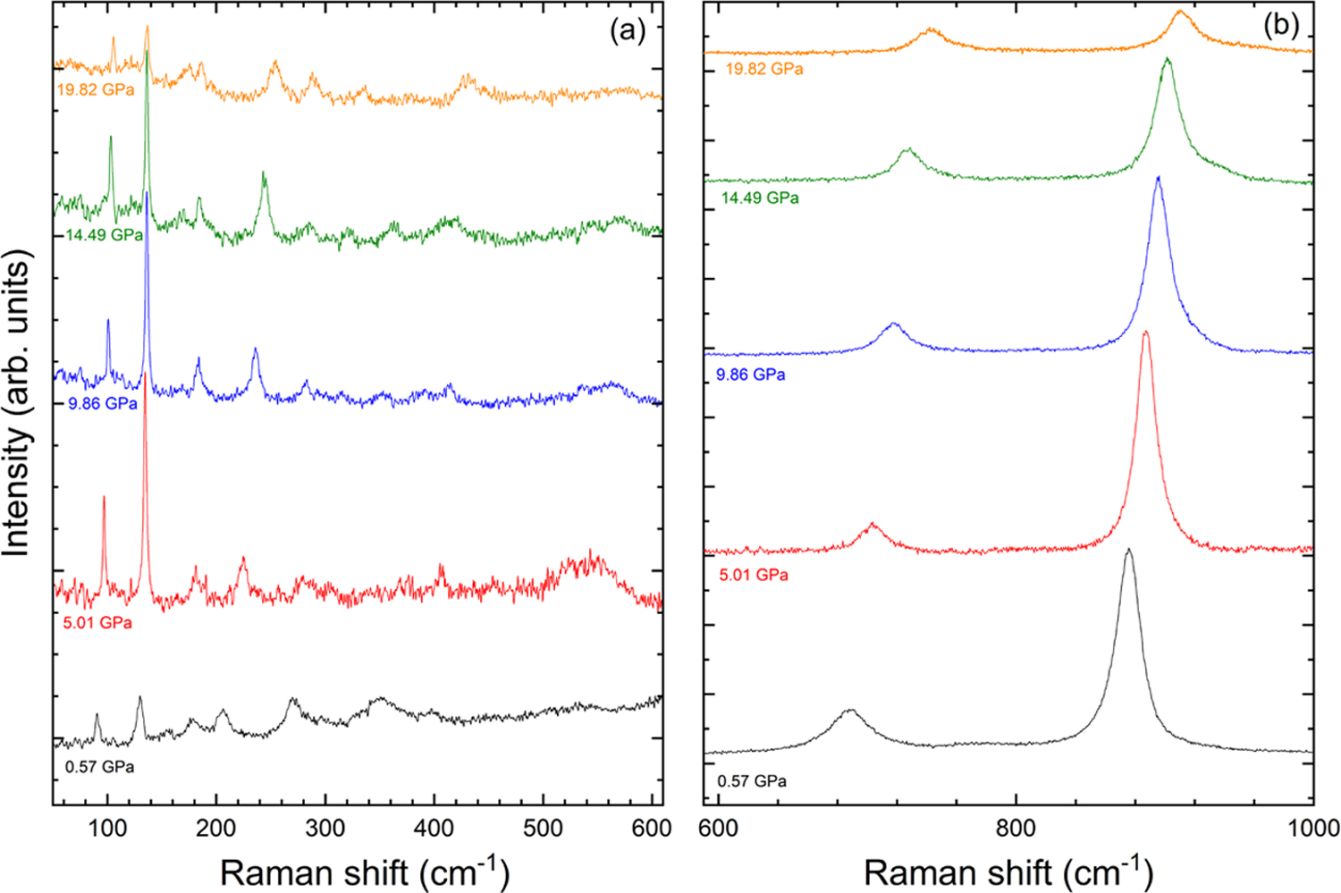
Raman spectra of natural mineral ferberite (FeWO_4_) at selected pressures. (a) The 50–600 cm^–1^ region. The spectra have been magnified five times to facilitate the identification of weak peaks. (b) The 600–1000 cm^–1^ region without magnification.

A selection of Raman spectra measured at different pressures is shown in Figure 6. The only changes observed in the spectra are a shift in the position of the modes and a decrease in their intensity. All spectra up to the highest pressure are compatible with the wolframite-type structure. We have observed the 18 modes only up to 2 GPa. The two weakest high-frequency modes cannot be distinguished from the background at this pressure. There are other modes that also cannot be detected up to the highest pressure (19.8 GPa), where only 12 Raman modes are observed.

**Figure 7:**
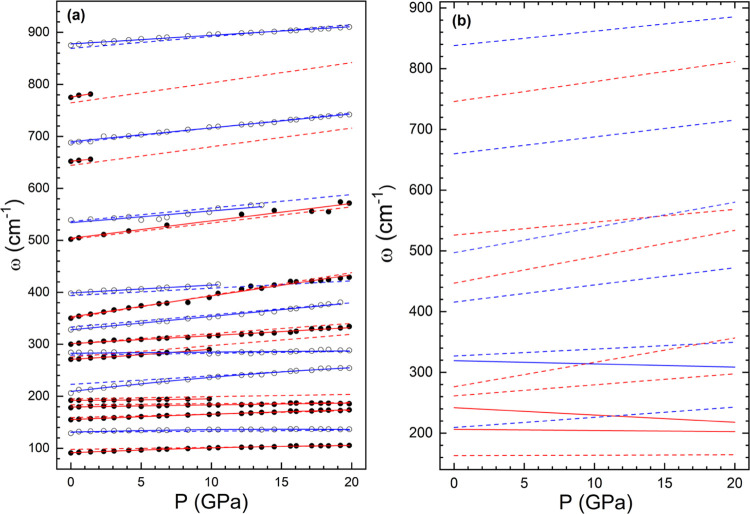
(a) Pressure dependence of the Raman-active modes in FeWO_4_. Black (empty) symbols represent the experimental B_g_ (A_g_) modes. Red (blue) solid lines are quadratic or linear fits to the experimental B_g_ (A_g_) modes. The dashed lines represent the results of calculations (the color code is the same as for experiments). (b) Calculated pressure dependence of IR-active modes. Red (blue) lines represent the B_u_ (A_u_) modes. Solid lines have been used to identify the modes which soften under compression.

The pressure dependence of the Raman-active modes of ferberite is plotted in Figure 7a. A few low-frequency modes follow a quadratic pressure dependence, and the rest follow a linear trend. All modes harden under compression. In Figure 7a, we also plot the results of the DFT calculations, which are in very good agreement with the experimental results. In Table 5, we summarize the wavenumber (ω), the pressure coefficients at zero pressure (dω/d*P*), and the Grüneisen parameter (, where ω_0_ is the wavenumber at zero pressure). The mode with the larger γ, i.e., the mode whose frequency varies more with the volume, is the B_g_ mode with wavenumber 350 cm^–1^. This is the internal bending mode of the WO_6_ octahedron. This is in agreement with the results reported for MgWO_4_ and MnWO_4_.^2,7^Figure 7a also shows that there are two phonon crossings between the A_g_ and B_g_ modes. One occurs at 5 GPa and the other at 15 GPa.

**Table 5:** Wavenumber (ω), Pressure Coefficient (dω/dP), and Grüneisen Parameter (γ) for Raman-Active and IR-Active Modes in FeWO_4_

mode	ω_exp_ (cm^–1^)	dω/d*P* (cm^–1^/Gpa)	γ	ω_DFT_ (cm^–1^)	dω/d*P* (cm^–1^/Gpa)	γ	mode	ω_DFT_ (cm^–1^)	dω/d*P* (cm^–1^/Gpa)	γ
B_g_	91	0.66	0.99	97.8	0.38	0.73	B_u_	163.0	0.07	0.10
A_g_	129	0.59	0.62	130.9	0.22	0.32	B_u_	206.3	–0.19	–0.17
B_g_	155	0.89	0.78	159.0	0.69	0.82	A_u_	209.2	1.68	1.45
B_g_	178	0.46	0.35	183.7	0.21	0.22	B_u_	241.9	–1.20	–0.99
B_g_	192	0.22	0.16	194.9	0.44	0.41	B_u_	261.2	1.82	1.27
A_g_	206	1.64	1.08	222.9	1.61	1.31	B_u_	276.1	4.03	2.48
B_g_	271	2.67	1.34	275.5	2.18	1.44	A_u_	319.2	–0.53	–0.32
A_g_	284	0.53	0.25	279.1	0.37	0.25	A_u_	326.8	1.14	0.66
B_g_	300	1.95	0.88	301.1	1.95	1.18	A_u_	415.6	2.83	1.23
A_g_	328	2.53	1.05	333.1	2.34	1.27	B_u_	446.9	4.35	1.71
B_g_	350	4.36	1.69	3502	4.38	2.18	A_u_	496.9	4.16	1.50
A_g_	398	0.59	0.20	393.7	1.41	0.67	B_u_	526.0	2.12	0.75
B_g_	502	3.13	0.85	502.1	3.11	1.13	A_u_	660.0	2.77	0.79
A_g_	539	1.95	0.49	536.3	2.59	0.90	B_u_	746.0	3.28	0.82
B_g_	648	3.33	0.69	644.1	3.59	1.02	A_u_	838.1	2.37	0.54
A_g_	688	2.87	0.57	686.8	2.92	0.79				
B_g_	775	3.91	0.69	764.4	3.87	0.94				
A_g_	875	1.95	0.30	869.2	2.28	0.50				

From the DFT calculations, we have obtained the frequency and symmetry assignment of the IR-active modes of FeWO_4_ as well as their pressure dependences. The results are summarized in Figure 7b and Table 5. They are presented for the sake of completeness and to help in mode assignment and identification in future IR absorption experiments. It can be observed that IR-active modes have a similar frequency distribution to Raman-active modes. In contrast to Raman-active modes, three IR-active modes soften with increasing pressure according to our calculations. The pressure dependence of IR modes has not yet been studied for other wolframites, so we do not know if the pressure-induced IR softening observed in FeWO_4_ is a fingerprint of this material or a common feature of the wolframite family. The IR mode with the largest Grüneisen parameter is the B_u_ mode with a wavenumber of 276.1 cm^–1^. Regarding phonon crossings and anticrossings, there are five phonon crossings and no phonon anticrossing predicted to occur up to 20 GPa, as shown in Figure 7a,b.

### 4.3 Optical Absorption Measurements

**Figure 8:**
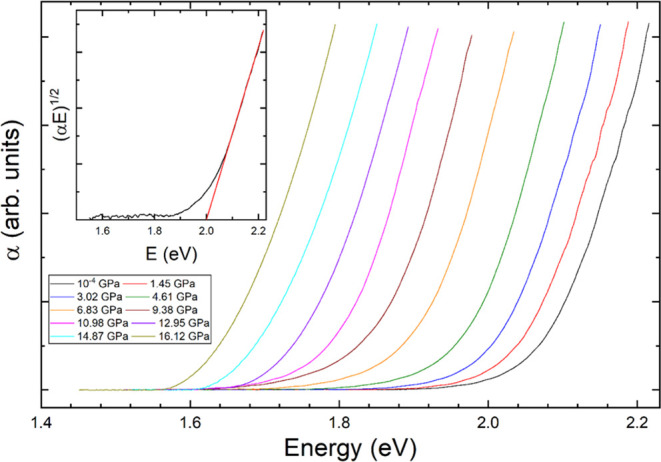
Optical absorption spectra of the natural mineral ferberite (FeWO_4_) measured at different pressures are indicated in the legend. The inset shows the Tauc plot used to determine the band gap energy at ambient pressure.

**Figure 9:**
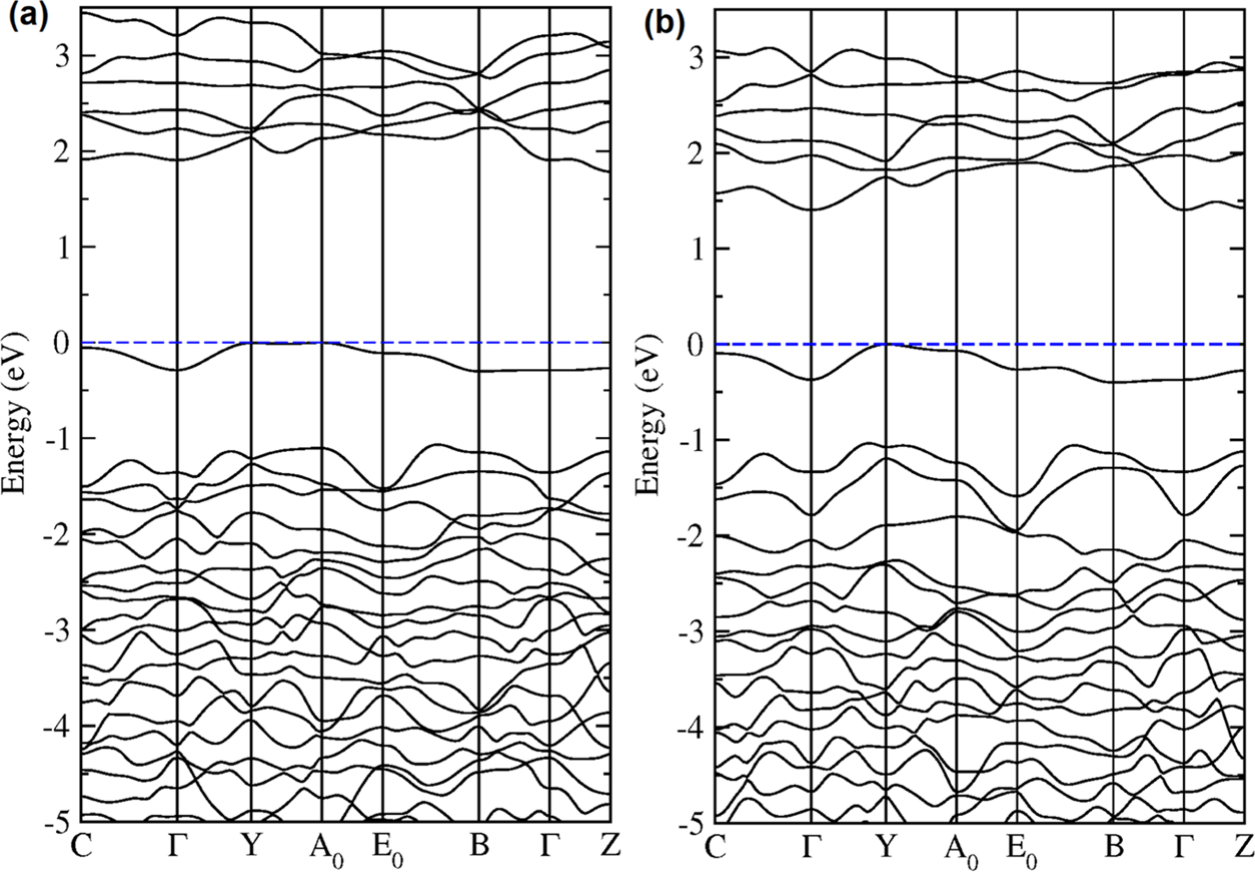
Calculated band structure of FeWO_4_ at (a) ambient pressure and (b) 13.4 GPa. The dashed blue line is the Fermi level, which has been set at 0 eV.

**Figure 10:**
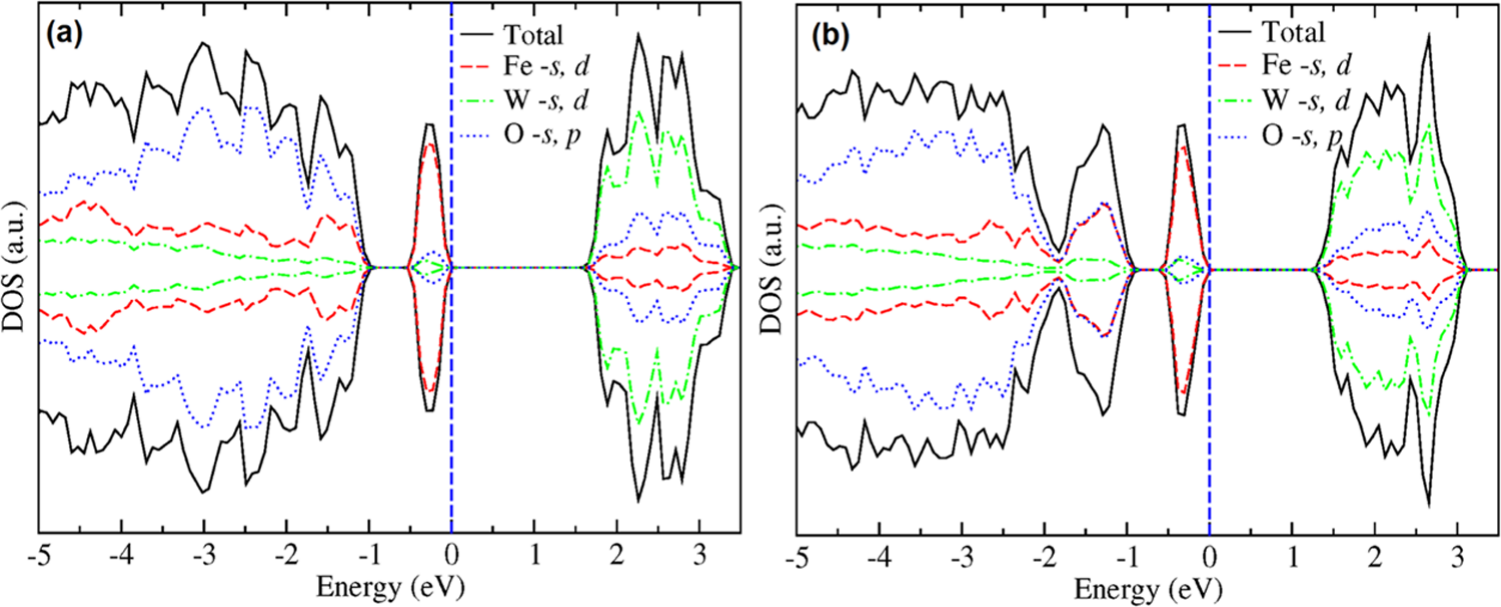
Electronic density of states of FeWO_4_ at (a) ambient pressure and (b) 13.4 GPa. The blue line is the Fermi level, which has been set at 0 eV.

To study the electronic properties of FeWO_4_ at ambient pressure and under compression, we performed optical absorption experiments as well as DFT simulations of the electronic band structure and density of states. The optical absorption spectrum of the natural mineral ferberite at different pressures is shown in Figure 8. The shape of the absorption edge supports an indirect band gap. We have also observed a typical Urbach-type^56^ exponential sub-band gap absorption, which is normally observed in tungstates and related ternary oxides.^57^ According to our simulations, the band gap of FeWO_4_ is indirect, in agreement with our experiments. The band structure is shown in Figure 9. The top of the valence band is at the Y point of the BZ and the bottom of the conduction band is at the Γ point of the BZ. DFT calculations give a band gap energy (*E*_g_) of 1.79 eV. Experimentally, we have determined the band gap energy by means of a Tauc analysis^58^ (see the inset of Figure 8); therefore, the reported value should be assumed as the lower limit value of *E*_g_.^59^ We obtained *E*_g_ = 2.00(5) eV, which is between the previously reported values.^10,12−14^ This means that the calculated band gap energy is slightly underestimated, but the underestimation, 0.2 eV, is within typical differences between DFT and experiments.^23,60^ However, this does not affect the determination of the pressure dependence of *E*_g_.^60^ In Figure 10, we present the electronic density of states, which shows that Fe states are the main contributors to the top of the valence band and hybridized W and O orbitals dominate the bottom of the conduction band.

**Figure 11:**
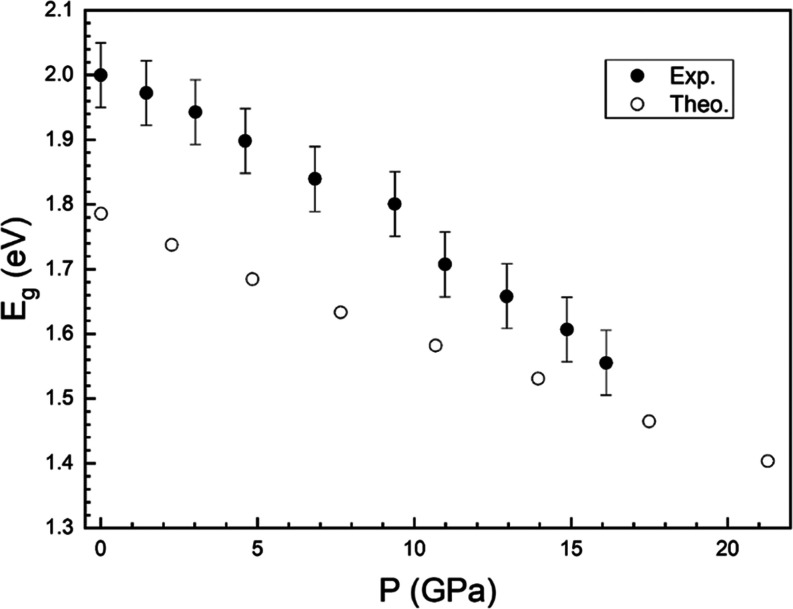
Pressure dependence of the band gap energy of FeWO_4_. Solid (empty) symbols are results from experiments (calculations).

The pressure dependence of the band gap energy from experiments and calculations is shown in Figure 11. Both methods give a qualitatively similar behavior, with the band gap closing at a rate of 25 and 20 meV/GPa according to experiments and calculations, respectively. This phenomenon is similar to the HP band gap closure reported for NiWO_4_,^19^ CoWO_4_,^22^ and MnWO_4_,^61^ and different from that found in CdWO_4_, ZnWO_4_, and MgWO_4_.^59^ The main reason for the band gap closure in FeWO_4_ at HP is the contribution of Fe orbitals to the top of the valence band. They move more quickly to higher energies under compression than the states at the bottom of the conduction band, causing the narrowing observed in *E*_g_. This can be seen in Figures 9 and 10 where the band structure and electronic density of states at different pressures are compared. In addition to reducing the band gap, the main change induced by pressure in the band structure is in the topology of the conduction band, which becomes more dispersive at high pressure. On the other hand, in the figure of the density of states, it can be seen that the orbital composition of the bands near the Fermi level is not modified under compression, but the Fe orbitals in the top of the valence band get closer to the bottom of the conduction band.

## 5 Conclusions

We report an experimental and theoretical study of the natural mineral ferberite (FeWO_4_). The crystal structure, Raman-active phonons, and band gap energy have been accurately characterized both experimentally and computationally at ambient pressure and under compression up to 20 GPa, and we have found them to be in good agreement. We found that the ambient pressure wolframite-type structure of FeWO_4_ does not undergo any phase transition up to 20 GPa. FeWO_4_ exhibits an indirect band gap of 2.00(5) eV at ambient pressure, which decreases at a rate of 25 meV/GPa similar to other magnetic AWO_4_ (A = Mn, Ni, and Co) wolframites. The pressure dependence of Raman-active and IR-active modes has been reported, with all Raman-active modes hardening with pressure, as it is characteristic of the AWO_4_ wolframite compounds, and several IR-active modes showing pressure-induced softening. In general, we have found that FeWO_4_ follows a similar trend to other wolframites, with the behavior of the electronic structure being particularly different from the nonmagnetic AWO_4_ (A = Mg, Zn, Cd) wolframites due to the valence band electrons from Fe.

## Data Availability

The data that support the findings of this study are available from the corresponding author upon reasonable request.

## Supporting Information Available

Crystallographic data (PDF)
