# Supplementary material for: Electronic, Vibrational, and Structural Properties of the Natural Mineral Ferberite (FeWO4): A High-Pressure Study
Source: Inorg Chem. 2024 Mar 30;63(15):6898–908. doi: 10.1021/acs.inorgchem.4c00345 (PMC11022173; doi:10.1021/acs.inorgchem.4c00345)
Supplement: Supplementary file 1 — ic4c00345_si_001.pdf [file ic4c00345_si_001.pdf]

Supplementary Information

Electronic, Vibrational, and Structural Properties of the Natural Mineral Ferberite ($FeWO_4$): A High-Pressure Study

Daniel Diaz-Anichtchenko,[†] Jesus E. Aviles-Coronado,[‡] Sinhué López-Moreno,[, §, ⊥] Robin Turnbull,[†] Francisco J. Manjón,[¶] Catalin Popescu,[‖] Daniel Errandonea[*,†]*

[†]Departamento de Física Aplicada-ICMUV, MALTA Consolider Team, Universidad de Valencia, Dr. Moliner 50, Burjassot, 46100 Valencia, Spain

[‡]División de Materiales Avanzados, IPICYT, Camino a la Presa de San José 2055 Col. Lomas 4a sección, San Luis Potosí 78216, México

[§]CONAHCYT - División de Materiales Avanzados, IPICYT, Camino a la Presa de San José 2055 Col. Lomas 4a sección, San Luis Potosí 78216, México

[⊥]Grupo de Ciencia e Ingeniería Computacionales - Centro Nacional de Supercómputo, IPICYT, Camino a la Presa de San José 2055 Col. Lomas 4a sección, San Luis Potosí 78216, México

[¶]Instituto de Diseño para la Fabricación y Producción Automatizada, MALTA Consolider Team, Universitat Politècnica de València, Camí de Vera s/n, 46022 València, Spain

[‖]CELLS-ALBA Synchrotron Light Facility, Cerdanyola, 08290 Barcelona, Spain

Crystallographic Data (Powder)
Source: MSPD beamline – ALBA synchrotron - X-ray 0.4246 Å
Chemical formula: $FeWO_4$
Formula weight: 303.68 g/mol
Temperature: 300 K
Pressure: ambient
Wavelength for constant wavelength or TOF: 0.4246 Å
Crystal system: monoclinic
Space group (No.) P2/c (No. 13)
a, *b*, *c*, α, β, γ: 4.74200 Å, 5.72200 Å, 4.97100 Å. 90.0000º, 90.1400º, 90.0000º
V (A_3): 134.881350 Å^3
Z = 2
d-space range: 1.247 – 6.935
χ_2: 1.98
R_p: 3.98%
R_{wp}: 6.33%
